# Environment consistently impact on aquaculture: The predominant source of residual pollutants in cultured Chinese mitten crab (*Eriocheir sinensis*) across China

**DOI:** 10.1016/j.heliyon.2024.e32418

**Published:** 2024-06-04

**Authors:** Longxiang Fang, Xi Chen, Limin Fan, Gengdong Hu, Liping Qiu, Chao Song, Yuwei Xie, John P. Giesy, Changbo Wang, Shunlong Meng

**Affiliations:** a Freshwater Fisheries Research Center, Chinese Academy of Fishery Sciences, Wuxi, 214081, China; b Laboratory of Quality & Safety Risk Assessment for Aquatic Products on Environmental Factors (Wuxi), Ministry of Agriculture and Rural Affairs, Wuxi, 214081, China; c Key Laboratory of Control of Quality and Safety for Aquatic Products, Ministry of Agriculture and Rural Affairs, Wuxi, 214081, China; d Key Laboratory of Freshwater Fisheries and Germplasm Resources Utilization, Ministry of Agriculture and Rural Affairs, Freshwater Fisheries Research Center, Chinese Academy of Fishery Sciences, Wuxi, 214081, China; e Huazhong Agriculture University, College of Fisheries, Wuhan, 430070, China; f Nanjing Institute of Environmental Science, Ministry of Ecology and Environment of China, Nanjing, 210042, China; g Key Laboratory of Pesticide Environmental Assessment and Pollution Control, Ministry of Ecology and Environment of China, Nanjing, 210042, China; h Department of Veterinary Biomedical Sciences, University of Saskatchewan, Saskatoon, Saskatchewan, S7N5B4, Canada; i Toxicology Centre, University of Saskatchewan, Saskatoon, Saskatchewan, S7N 5B3, Canada; j Department of Integrative Biology, Michigan State University, East Lansing, MI, 48895, USA; k Department of Environmental Sciences, Baylor University, Waco, TX, 76798-7266, USA; l Kunshan Fisheries Technology Extension Center, Kunshan, 215300, China

**Keywords:** Pollutants, Food, Priority control, Sources, Asia

## Abstract

Advancements in monitoring and operation of aquaculture environments has minimized the concentrations of some residual pollutants in cultured aquatic products. However, currently most aquatic products are “farmed”, and relationships among residual pollutants in tissues of crabs were still unclear. In this study, 64 typical pollutants, including 25 antibiotics, 15 metal, 23 organochlorine pesticides, and one dioxin-like compound inducing hydrocarbon-receptor (AHR) activity were measured in Chinese mitten crab (*Eriocheir Sinensis*) risks of consumption assessed and ranked. The superposition of properties including severity and relative potency of effects and parameters describing persistence and exposure along with rates of usage and identification of groups most likely to be exposed were assessed in combination to rank likelihood of dietary exposure and probabilities of adverse effects for each contaminant. The results indicated that the total scores per pollutants found that Cadmium (Cd), Heptachlor epoxide (HEPE), dioxin TEQ exhibited the greatest scores and explained the severity of dietary risk, while source analysis found that the three main pollutants resulted from the ambient environment and are not due to specific aquaculture processes. In summary, environment is still the predominant source of residual pollutants in cultured Chinese mitten crab across China.

## Highlights

• The consumption risk of 64 typical pollutants in crab were assessed and ranked.
• Cd, HEPE, Dioxin showed highest dietary risk should be priority monitored.
• Environment is still the predominant source of residual pollutants.

## 1 Introduction

Aquaculture is one of the most efficient and reliable solutions to ensure food security, but there are concerns about uses of veterinary pharmaceuticals. Aquaculture products provide nearly 20 % of the daily animal protein intake for approximately 3.1 billion people, worldwide [1,2]. According to the Food and Agriculture Organization of the United Nations (FAO), aquaculture is the fastest growing segment of production of food worldwide, growing at an average annual rate of 8 % over the past 30 years, even reaching a record great of 101.1 million metric tons, which in 2014 was more than the total wild catch. Yields of cultured aquatic products in China have continuously increased and now account for more than 50 % of total production worldwide [3,4]. However, except for meeting the global demand for food, some public issues regarding food safety have remained a concern [5]. Local governments reported that dozens of events related to the quality and safety of aquatic products occur in China each year [6,7]. Some farming inputs, containing antibiotics, can result in residues of these pharmaceuticals in edible parts, with concentrations ranging from several μg/kg to hundreds of μg/kg, and in some cases exceed the maximum residue limit (MRL) established by various jurisdictions [8,9]. Simultaneously, multi contaminants, such as metals, pesticides, or persistent organic pollutants (POPs) from aquaculture environment, can be accumulated into food chain due to bioaccumulation and biomagnification [7,10]. However, within mixtures of contaminants, it was still unknown which constituents and their sources pose the most significant risks to health of humans who consume aquatic foods.

Fish ponds which are the most frequently used cultural mode for high density aquaculture in China, can become contaminated by point and non-point urban and rural sources. Although fishponds are semi-closed artificial ecosystem, various contaminants can enter via applications of pharmaceutical drugs, overland runoff, input of feeds, and environmental residue [11,12]. As one of China's favorite crustacean species to eat, the Chinese mitten crab (*Eriocheir Sinensis*, which hereafter is simplified to just crab) extensively cultured with more than 6 million hectares [2]. Additionally, due to the benthic habits of crabs and physical properties, specific contaminants, such as metals and organic pesticides, can be more likely to residue in the edible parts of the crab [13]. Based on results of previous research, Cadmium (Cd) and Arsenic (As) were detected in all samples, with mean concentrations of 0.15 and 0.96 μg/kg (wet mass), respectively. Organic contaminants, such as, pesticides, were also detected. The results indicated that they might be easily bioaccumulated into brown meat, a general term for the gonad and hepatopancreas, because of relatively great high fat content (>45 %) of those tissues [14].

Quantification of dietary risk is challenging to evaluate the safety extent of contaminants in aquatic products [9,15]. Initially, the dietary or ecological risks were quantified using a probabilistic risk assessment approach, and the result was exhibited as the joint probabilities between the exposure route and the hazard effects of specific contaminants. Subsequently, researchers developed the risk-based approach for cost-effective use of food safety monitoring [16,17]. Several factors represent each step of the assessment of exposure and hazard, including usage, evidence of residues, severity of effects and toxic potency [6,18]. Until now, this dietary risk-based model has been successfully applied to target the most susceptible antibiotics in cultured aquatic products or other food animals and pesticides in the dietary intake of celery in China [19,20]. However, what was dietary risk from consumption of crab and which types of contaminants were more urgently in need of monitoring?

In the present study, based on the calculated ranking results, we proposed the plan for monitoring target contaminants that cause most dietary risk as precision monitoring, which helps to allocate time, labor, and financial saving when the government conducts supervision over the quality and safety of aquatic products. Sixty-four types of contaminants caused by fishery drugs, overland runoff, the input of feeds, and environmental residue, were detected in the collected cultured crab samples.

The specific objectives of this study were to [1]: rank the dietary risk of each contaminant, mining the most severe one [2]; establish a list of priority control of contaminants for guiding the consumption choices of cultured crab [3]; explore the main sources of greatest risk pollutants in crabs.

## 2 Materials and methods

### 2.1 Chemicals

In this study, due to living habits and physical properties, 64 candidate contaminants or chemicals that were likely accumulated in the edible parts of cultured crabs were selected, including 25 antibiotics, 15 metal, 23 organochlorine pesticides, and one dioxin-like compound inducing hydrocarbon-receptor (AHR) activity, referred to published articles [14,18,20]. Authentic standards with more than 98 % purity for chemicals were supplied by Dr. E. Horn GmbH company and Shanghai Anpel Experimental Technology Co., LTD., (Table S1).

### 2.2 Sampling strategy and samples

A total of 54 Chinese mitten crab (*Eriocheir sinensis*) were collected from Jiangsu Province, the province was not only the main production area, that contributed to approximately 50 percent of total yields in China, but also a vital market. With the 54 uniformly distributed sampling sites selected, thirty individuals crabs, half male and half female were collected and total edible parts, including brown meat (a general call of hepatopancreas and gonad) and white meat (muscle in legs and chest of crustaceans) were removed. Total edible parts were blended into one sample. Fifty-four samples were collected and stored at −20 °C until further analysis. All animal procedures were performed in accordance with the fishery resource management principles in China that were approved to and agreed upon by the Ethics Committee of the Freshwater Research Center, CAFS, CAFS-20200912-22.

### 2.3 The analysis of residual contaminants in the total edible part of Chinese mitten crab

Antibiotics detected in this study included the category sulfonamides and quinolones, which are permitted and the most widely used fishery drugs in aquaculture. Antibiotics were extracted by use of a QuEChERs method and then identified and quantified by use of LC-MS/MS [20]. Residual metals in crab were digested into solution before analysis in ICP-MS [14]. An accelerated solvent extraction followed by Florisil SPE column's purification was used to determine OCPs [18]. Subsequently, the elution containing targeting contaminants was transferred to GC-ECD for analysis. The data on the above three types of contaminants have been published in recent articles written by our research group.

The residual dioxin equivalents (TEQ) in crab was expressed by dioxin-like AHR activity, which was determined in vitro by use of the H4IIE-*Luc-*transactivation bioassay [21]. In brief, the total edible part was ASE extracted and purified by silica contained SPE column (Anpel, 6 g/3 mL) and the eluate was stored at −20 °C until assayed. H4IIE-*Luc* cells were rat hepatoma cells with a luciferase reporter gene stably transfected. Luciferase assays were conducted after exposure and calibrated by standard instrumental methods. Limit of quantification (LOQ), which was calculated by use of three replicate analyses near the LOQ, was 1.2 pg/g, wm.

### 2.4 Ranking protocol for prioritizing dietary risk of each contaminant

**Fig. 1:**
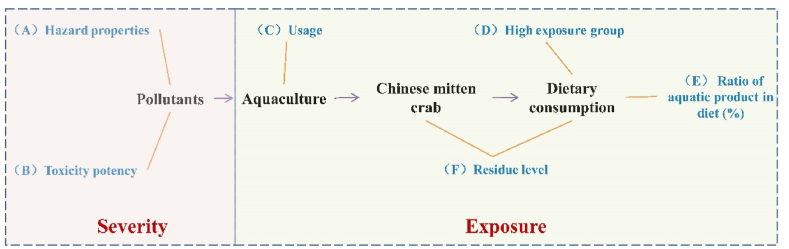
The ranking principle of dietary risk with residual contaminant in Chinese mitten crab (*Eriocheir Sinensis*), is defined as the superposition of severity properties and exposure routes. Details containing A: hazard properties; B: toxicity potency; C: usage; D: high exposure groups; E: ratio of aquatic product in the diet; F: residue level.

The dietary risk of residual contaminants in crab is defined by use of a combination of properties and exposure routes (Fig. 1) (Equation (1)). The principle of constructing this model is based on the probability that contaminants will gradually enter the human body from the “aquaculture-consumption” pathway [22].

(1) Equation1:TS=(A+B)×(C+D+E)×F

where TS is the total score, calculated based upon multiple individual scores, including A: hazard properties; B: toxicity potency; C: usage; D: exposure groups; E: ratio of aquatic product in the diet; F: residue level. Herein, the dietary risk was ranked by comparing the TS value of each contaminant [8]:

Among them, the inherent toxicity of contaminants is characterized by (A) hazard properties and (B) toxic potency. The hazard properties refer to contaminant's toxicity to human health at different levels caused by their inherent molecular structure or physical/chemical properties [23]. For example, if contaminants have been shown to have a definite carcinogenic effect on the human body and its mechanism is clarified, the score is the greatest; the toxicity potency value is the acceptable allowable intake (ADI). For exposure route, it is mainly characterized by four factors, one of which is whether contaminants are used during culturing of crab, that is, (C) the usage; Second, (D) whether there are great exposure groups; Third, the proportion of crab in total dietary consumption, that is, (E) ratio of aquatic product in diet (dietary proportion); Fourth, the degree of contaminants residues in crab is (F) residue level. The scores attributed to various of risk factors were shown in (Table S2).

### 2.5 Data analysis

The plot was drawn by Microsoft Excel 2007. The data were expressed by mean with standard deviation, and all statistical analyses were conducted by JMP 16.0 with a significant level of 0.05.

## 3 Results and discussions

**Fig. 2:**
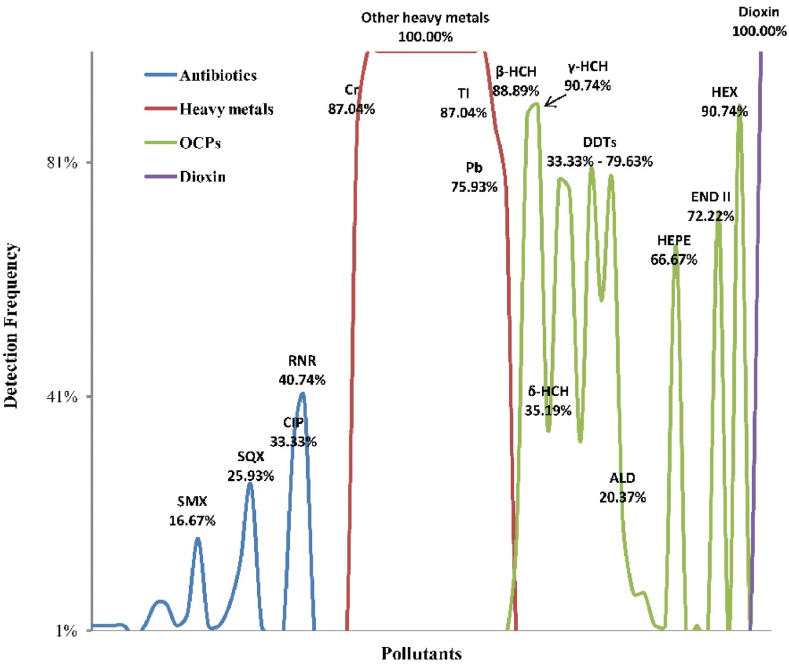
The detection frequencies of 64 contaminants (including 25 antibiotics, 15 heavy metals, 23 organochlorine pesticides, and one dioxin-like aromatic hydrocarbon receptor (AHR) activity) expressed in the crab edible parts were shown as below. Only pollutants with relatively high detection frequencies (more than 10 %) were counted.

**Fig. 3:**
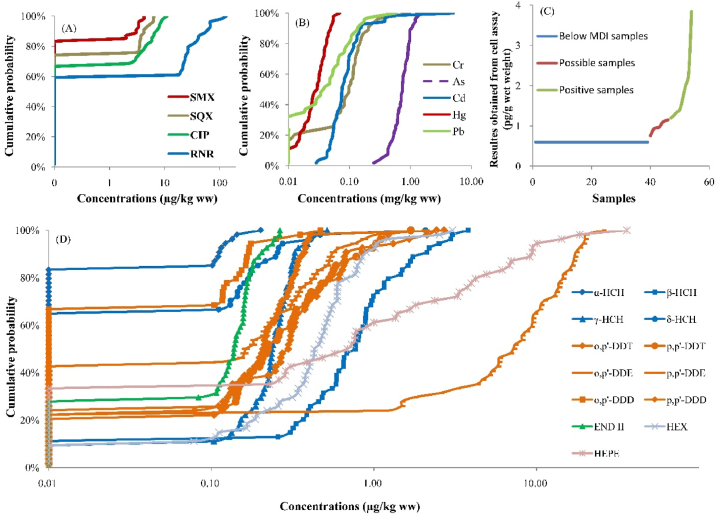
Distribution of the total concentrations with 64 contaminants (retain main substances) expressed in crab samples. A was the cumulative probability of antibiotics concentrations, B was the cumulative probability of heavy metals concentrations, C was the results obtained from cell assay and D was the cumulative probability of organochlorine pesticides concentrations.

Frequencies of detection and total concentration of 64 contaminants in edible parts of crab are Fig. 2, Fig. 3, these including 25 antibiotics, 15 metals, 23 organochlorine pesticides, and one dioxin-like aromatic hydrocarbon receptor (AHR) activity. The 64 pollutants were detected in a variety of samples. These pollutants with widely applied, environmentally persistent, and lipid solubility can be biomagnified in food webs, and the dietary risk in mitten crab deserve more attention [24]. The aim was to determine which pollutant is most important to monitor for the surveillance of mitten crab dietary risk in future.

### 3.1 The exposure routes and residual concentrations of 64 pollutants in Chinese mitten crab

#### 3.1.1 RNR was the most frequent detected antibiotics with the greatest concentrations

RNR was detected in crabs at the greatest frequencies and concentrations in crabs relative to other antibiotics. Among monitored antibiotics, 18 of 25 monitored antibiotics were found in crab with frequencies of detection ranging from 1.9% to 40.7 %. RNR, CIP, SQX and SMX were the main types of antibiotics with detection frequencies of 40.7 %, 33.3 %, 25.9 % and 16.7 %, respectively (Fig. 2). In this study, concentration of RNR also among the greatest concentrations among the 25 targets, the maximum concentration was 132.9 μg/kg wm, which exceede the maximum residue limit (MRL) of 100 μg/kg, wm (Fig. 3A). Similarly, quinolones were frequently detected in the cultured fish samples in Haihe River and Pearl River Delta, while RNR was detected at the greatest frequency, which was 40 % [7,9]. When concentrations of eight antibiotics were examined in fishes, RNR explained the greatest antibiotic concentration [25]. Greater concentrations of residues is caused by the wide use of RNR during aquaculture [13,26]. Notably, CIP was still detected although it was banned in Chinese aquaculture. The reasons for the detection of CIP in crabs might be that, CIP was the main metabolite of RNR, and the presence of CIP in aquatic products were dependent on degradation of RNR [8,20]. Quinolones are prohibited in aquaculture by the Chinese Ministry of Agriculture and some other international organizations [4,8].

#### 3.1.2 Concentrations of metals in crabs

Several metals, including Manganese (Mn), Cobalt (Co), Nickel (Ni), Gallium (Ga), Arsenic (As), Selenium (Se), Rubidium (Rb), Argentum (Ag) and Cadmium (Cd), except for Chromium (Cr) (87.04 %), Thallium (Tl) (87.04 %), and lead (Pb) (75.93 %) were detected in all samples (Fig. 2). Among these, Cd, As, Cr, Hg and Pb were the non-essential metals, these metals could be bioaccumulated via food web, and have proven to be the greatest threat to health of humans after dietary consumption [27]. Moreover, maximum concentrations of other metals, such as Mn, Co, Ni and Ga were detected, but occurred at concentrations much less than their respective MRLs set by the Chinese Health Ministry [28] and European Commission [29].

Concentrations of Cd ranged from 0.03 to 5.02 mg/kg wm, 53 (98.15 %) of the crab samples did not exceed the Chinese MRL of 0.5 mg/kg, wm (Fig. 3B) [30]. Mean concentrations of Cd observed in crab were less than those reported for fish sample (2.17–4 mg/kg) from Beyşehir Lake in Turkey and fish sample (0.26 mg/kg) from Miankaleh Lake, respectively [31,32]. Accumulation of Cd in the human body can cause chronic poisoning, induce skeletal damage and functions of kidney or reproduction system [33,34].

Total concentrations of As in crab ranged from 0.25 to 1.49 mg/kg, wm (Fig. 3B), which was comparable to concentrations observed in fishes (0.65–1.98 mg/kg) in freshwater ecosystems from Sam Roque Lake in Argentina [16,35]. However, no MRL has been set for total As in aquaculture products. The toxicity of elemental As is relatively small among samples of crab, while organic arsenics are highly toxic [13,15].

#### 3.1.3 Residual OCPs in crabs

Nineteen of 23 (82.61 %) OCPs, except for α-CHL, END I, Me DDT and MIR, were detected in crab. The greater detection frequencies of chlorobenzene OCPs were for HCHs (*γ*-HCH up to 90.74 %) and DDTs (*p,p'*-DDD up to 79.63 %), with concentrations ranging from <LOQ-3.80 and <LOQ-25.52 μg/kg (Fig. 2, Fig. 3D), respectively. Previously, HCHs and DDTs were confirmed to be the largest contributors [36,37]. These results were comparable with concentrations reported from other regions and basins in China [38]. For instance, fishes collected from the Qiantang River had concentrations of HCHs and DDTs of 1.9–5.9 and 2.7–133.5 μg/kg, and were the greatest contributors to total concentrations of OCPs [6]. Concentrations of OCPs in biota are positively correlated with lipid content [13]. In mitten crab, there is approximately 40 % of brown meat, which is lipid rich and greater than that of fishes [18]. Mitten crabs are benthic and thus more likely to be exposed to OCPs [3]. Furthermore, DDTs have been banned since the 1980s, but they are still approved for some applications [[39], [40], [41]]. A greater proportion of DDTs were detected in a few samples, indicating recent of DDT input.

HEPE were the chlorinated, alicyclic OCPs which occurred at the greatest concentrations. Concentrations of HEPE great as 36.0 μg/kg wm were measured in crab, but this was skewed because a few samples had greater concentrations [42]. Although the use of HEPE has been strictly restricted in China [43], some countries have not attached attention to this [44]. For instance, a large amount of HEPE has been detected in the Three Gorges Reservoir in China (fish and sediments), as well as in the aquatic environment of Côte d’Ivoire [5], therefore, the possible long-distance transportation way of HEPE residues can via atmosphere [19]. Additionally, the greatest mean concentration of compounds was HEPE, this indicates that the focus of OCPs residues cannot only stay on HCHs and DDTs, and chlorinated alicyclic OCPs need more attention [37,45].

#### 3.1.4 Nonnegligible residual dioxin in crabs

Concentrations of residual dioxin equivalents in crab ranged from 0.75 to 3.84 pg TEQ/g. Dioxin have strong carcinogenicity (10 pg/g to cause liver cancer in rats) and a variety of toxic effects including environmental endocrine disrupting effects at very low doses (Fig. 3C) [21].

Compounds that are dioxin-like and principle via the Arylhydrocarbon receptor (AhR) are synthetic chemicals that are produced by many industrial processes including synthesis of chemicals and as biproducts of combustion and in some chlor-alkali processes [21,46].

### 3.2 The severity of main contaminants with evident exposure routes

**Fig. 4:**
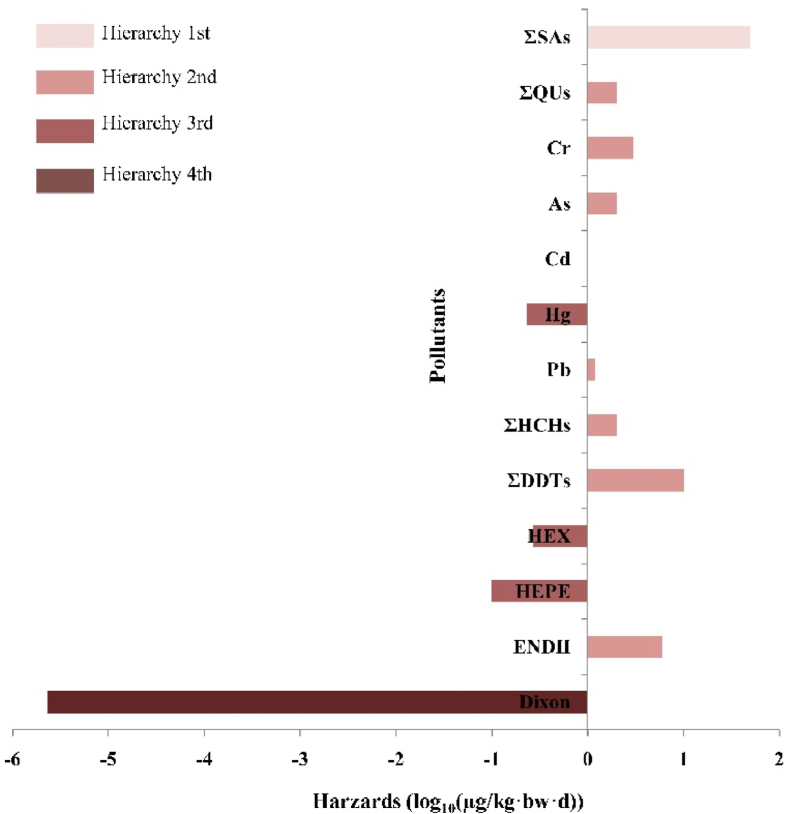
The toxicity potency of 64 pollutants (retain the main substance) was derived from the acceptable daily intake (ADI) value of each pollutant, data were transformed by log10 ranged from −5.64 to 1.70. The hazards of selected pollutants were in four degrees, with smaller ADI value, the pollutant explained higher toxic.

**Fig. 5:**
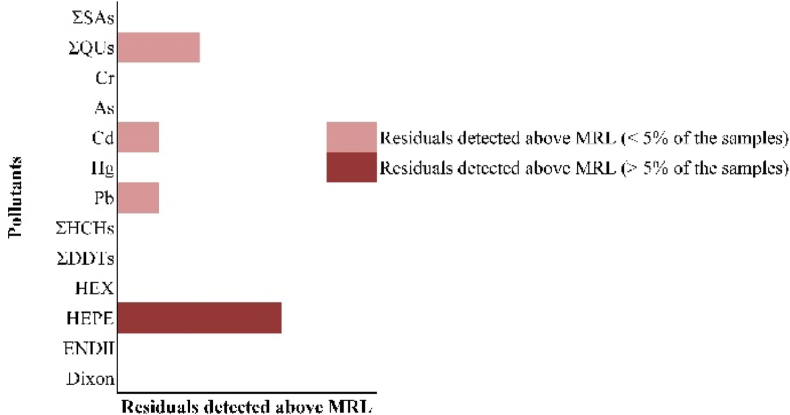
Among the crab samples, with the total concentrations of each pollutant, according to the maximum residual limit (MRLs) derived from relevant standard, <5 % of the sample and >5 % of the sample residual detected above the MRLs were counted.

The ADI value was log10 transformed as severity to facilitate unified comparison for detected contaminant. The greatest hazard quotient (HQ) was for TEQ (−5.64) in Hierarchy 4, represents the most severe toxic, followed by Hg, HEX and HEPE in Hierarchy 3, ENDII, ΣDDTs, ΣHCHs, Pb, Cd, As, Cr and ΣQUs in Hierarchy 2. And the greatest value was ΣSAs (1.6) in Hierarchy 1, indicating that ΣSAs was likely to cause de minimis toxicity (Fig. 4). The hierarchy of major contaminants was consistent with their toxic potency. Furthermore (Fig. 5), with ΣQUs, Cd, and Pb residuals, <5 % of samples of edible tissues of crab exceeded the MRL. As for HEPE, >5 % of the crab samples residuals detected above MRL. The concentration of other contaminants was all less than the MRL.

Dioxin-like chemicals are a class of toxic substances, and has toxic potency that is 130-fold than cyanide and 900-fold greater than As [21,46]. Thus, very small concentrations of TEQ can have large adverse effect on animals, and there is also strong carcinogenicity (the dose of 10 pg/g to cause liver cancer in rats) [10]. These substances are neither produced nor used for any beneficial purpose, but rather are by-products of combustion and various industrial processes. The main sources of dioxins in the environment were via evaporation caused by the use of chlorophenols in wood preservation, schistosomiasis prevention and emissions from incineration industries [2,10].

In aquaculture systems, OCPs can act as endocrine disruptors, causing damage to reproduction, developmental and growth of organisms and humans [36,40]. Importantly, the Chinese government has taken effective measures to limit the use of DDT and HCH in crop cultivation. However, due to the great environmental persistence, OCPs can persist in aquatic environments for many years [39]. Therefore, their residues and metabolic forms can still be detected in aquatic biota.

Among these pollutants, antibiotics showed the minimal toxicity. Antibiotics are natural or synthetic drugs that have the ability to kill bacteria or inhibit their growth [2,25]. They are commonly used in animals to prevent and treat *Aeromonas*, *Pseudomonas* and *Cytophaga pathogens* by blocking bacterial DNA replication, and some are also used as growth promoters in farm animals [8]. Continued antibiotic use can promote antibiotic resistance genes in bacterial populations in the environment, humans, and farm animals [47]. In addition, antibiotics residues in farm animals can pose a risk to people who consumes them. Except for dietary risks, there are also couple with environmental hazards, and primary productivity plays an important role in aquaculture systems, such providing food for fish [20,47]. However, increased concentrations of antibiotics in water have hindered the growth of green algae. And certain classes of antibiotics, such as sulfonamides, can inhibit denitrification in biogeochemical cycles, further stimulating the release of nitrous oxide (N_2_O). Thus, the use of antibiotics can exacerbate eutrophication and the greenhouse effects antibiotics usage [2,8].

### 3.3 Dietary risk ranking for priority control of contaminants influencing consumption of crab

The scores for factors A, B, C, D, E and F were calculated based on our experiment (Table S3). Hazard properties of contaminants in crab samples were used to characterize the score for A. Dioxin and As exposure have been associated with neurodevelopment and cancer risk, proved to have a definite carcinogenic effect on the human body. Accordingly, these two compounds were assigned the greatest score [6], followed by Cd and HEPE [5], ΣSAs and ΣQUs [4]. The rank of toxicity potency value was Dixon > Hg > HEX > HEPE > ΣQUs, Cr, As, Cd, Pb, ΣHCHs, ΣDDTs and END II>ΣSAs. Dioxin posed the greatest toxicity risk, therefore it scored a 6. By contrast, ΣSAs was the least toxic, so it was assigned a score of 0.

Scores for the factors C, D, E and F were calculated based on exposure route. C represented the drug usage in crab farming. Statistic showed that of all fishery medicines, antibiotics are the most important and widely used in the production of aquatic products to preventing pathogens [20]. About 100,000 t of antibiotics are used annually in the production of food animals in China, including aquatic products [34]. Accordingly, the antibiotics of ΣSAs and ΣQUs were assigned great scores. Other contaminants were not used in aquaculture and these were assigned a score of zero. D was used to describe whether there are great exposure groups. E stands for consumption data, according to the survey of China Food Consumption Database, the estimated fish consumption of residents in the Tai Lake region is 0.0627 kg fish wet weight per day [15]. As a result, all contaminants are assigned an E score of 1. The presence of residual contaminants in crab sample was characterized as a score for factor F. HEPE, followed by ΣQUs, Cd, and Pb were the main types of residual contaminants. Using MRL, these residual contaminants are given great scores.

**Fig. 6:**
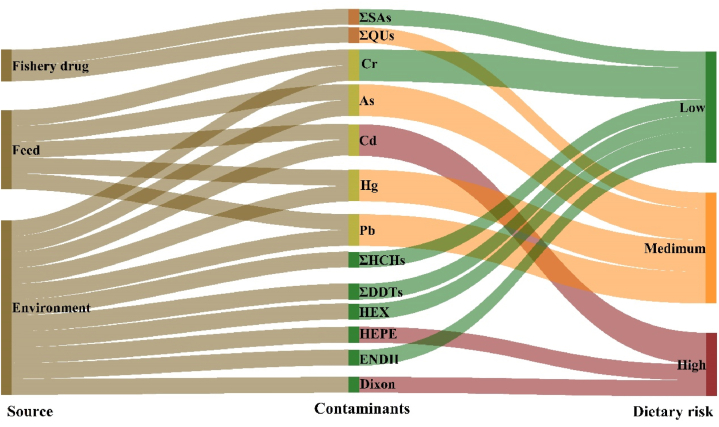
Sankey diagram of dietary risk ranking with detected pollutants. Including the pollutant source refer to the published reports.

The total scores (TS) of dietary risks for each contaminant was calculated from the scores for each factor, ranging from 20 to 84 (Fig. 6). The scores of Cd, HEPE and Dixon >70, indicating they posed the greatest toxicity. Followed by ΣQUs, Pb, As and Hg posed a medium toxicity, and the scores ranged from 42 to 60. Meanwhile, ΣHCHs, ΣDDTs, HEX, ΣSAs, ENDII and Cr explained a medium toxicity, the scores below 42. Using a risk ranking approach, it was found that Cd, HEPE and Dioxin posed the greatest dietary risk of all 64 selected pollutants, mainly due to the great toxicity of their hazard properties.

## 4 Conclusions

In studies, 64 typical residual contaminants in crab samples were assessed separately according to category. Here, a risk ranking approach was applied in a case study surrounding Jiangsu province, and the most important contaminant as Cd, HEPE and Dioxin posed the greatest dietary risk, and these pollutants should be priority selected for future surveillance. The main entry routes of these three pollutants into aquaculture systems via environment, the results indicated that the most influenced contaminant in aquaculture products were from the environment. In conclusion, from ancient times to the present, from fishing to aquaculture, the way to obtain aquatic products has changed, but the main source of pollutants in aquatic products has remained the same.

## Ethics declarations

All animal procedures were performed in accordance with the fishery resource management principles in China that were approved to and agreed upon by the Ethics Committee of the Freshwater Research Center, CAFS, CAFS-20200912-22.

## Funding

This work was supported by the 10.13039/100017837Wuxi Science and Technology Development Fund (2021162) and (K20231029), 10.13039/501100012453China Agricultural Research System (CARS-46) and Kunshan Yangcheng Lake Crab Industrial Research Institute (202105). Y.W. Xie is supported by by the Central Scientific Research Projects for Public Welfare Research Institutes. 10.13039/501100012166National Key Research and Development Program (Grant No. 2022YFC3902103).

## Data availability statement

The data that support the findings of this study are available from the corresponding author, [Chao Song], upon reasonable request.

## CRediT authorship contribution statement

**Longxiang Fang:** Methodology, Investigation. **Xi Chen:** Investigation. **Limin Fan:** Formal analysis. **Gengdong Hu:** Formal analysis. **Liping Qiu:** Formal analysis. **Chao Song:** Funding acquisition. **Yuwei Xie:** Funding acquisition. **John P. Giesy:** Funding acquisition. **Changbo Wang:** Supervision. **Shunlong Meng:** Supervision.

## Declaration of competing interest

The authors declare that they have no known competing financial interests or personal relationships that could have appeared to influence the work reported in this paper.
