# Supplementary material for: Environment consistently impact on aquaculture: The predominant source of residual pollutants in cultured Chinese mitten crab (Eriocheir sinensis) across China
Source: Heliyon. 2024 Jun 4;10(11):e32418. doi: 10.1016/j.heliyon.2024.e32418 (PMC11200344; doi:10.1016/j.heliyon.2024.e32418)
Supplement: Multimedia component 1 [file mmc1.docx]

Table S1 Due to living habits of Chinese mitten crab (*Eriocheir Sinensis*), and refer to the physical properties of pollutants, 64 candidate contaminants or chemicals (including 25 antibiotics, 15 heavy metals, 23 [organochlorine](javascript:;) [pesticide](javascript:;)s, and one dioxin-like [aromatic](javascript:;) [hydrocarbon](javascript:;) [receptor](javascript:;) (AHR) activity) that were likely accumulated in the edible parts of cultured crabs were selected. The chemical information, abbreviations, CAS number and origin of these compounds in aquaculture were shown as below.

| Serial number | Categories | Pollutant | Abbreviation | CAS | MRL | Standard | Reference |
| --- | --- | --- | --- | --- | --- | --- | --- |
| 1 | Antibiotics | Sulfaguanidine | SG | 57-67-0 | 100 μg/kg | The Announcement (The 235th) of Ministry of Agriculture (1) | Feed (2) |
| 2 |  | Sulfacetamide | SAA | 144-80-9 |  |  |  |
| 3 |  | Sulfadiazine | SDZ | 68-35-9 |  |  |  |
| 4 |  | Sulfamethazine | SDD | 57-68-1 |  |  |  |
| 5 |  | Sulfathiazole | SAT | 72-14-0 |  |  |  |
| 6 |  | Sulfamerazine | SMR | 127-79-7 |  |  |  |
| 7 |  | Sulfamoxole | SMO | 729-99-7 |  |  |  |
| 8 |  | Sulfameter | SMD | 651-06-9 |  |  |  |
| 9 |  | Sulfisomidine | SIM | 515-64-0 |  |  |  |
| 10 |  | Sulfamethoxypyridazine | SMP | 80-35-3 |  |  |  |
| 11 |  | Sulfamethoxazole | SMX | 723-46-6 |  |  |  |
| 12 |  | Sulfamonomethoxine | SMM | 1220-83-3 |  |  |  |
| 13 |  | Sulfadoxine | SDX | 2447-57-6 |  |  |  |
| 14 |  | Sulfisoxazole | SIZ | 127-69-5 |  |  |  |
| 15 |  | Sulfadimethoxine | SDM | 122-11-2 |  |  |  |
| 16 |  | Sulfaquinoxaline | SQX | 59-40-5 |  |  |  |
| 17 |  | Sulfachloropyridazine | SPDZ | 80-32-0 |  |  |  |
| 18 |  | Ofloxacin | OFL | 82419-36-1 | 100 μg/kg |  |  |
| 19 |  | Pefloxacin | PEF | 149676-40-4 |  |  |  |
| 20 |  | Ciproflxoacin | CIP | 85721-33-1 |  |  |  |
| 21 |  | Enrofloxacin | RNR | 93106-60-6 |  |  |  |
| 22 |  | Lomefloxacin | LOM | 98079-51-7 |  |  |  |
| 23 |  | Orbifloxacin | ORB | 113617-63-3 |  |  |  |
| 24 |  | Sarafloxacin | SAR | 91296-87-6 |  |  |  |
| 25 |  | Sparfloxacin | SPA | 110871-86-8 |  |  |  |
| 26 | Heavy metals | Chromium | Cr | 19498-56-7 | 2.0 mg/kg ww | National food safety standards Limits of contaminants in food (GB 2762-2012) (3) | Feed, Environment (4) |
| 27 |  | Manganese | Mn | [7439-96-5](http://www.baidu.com/link?url=QGSIzqy2jFkyVu4iDrenGSJW4ikezEbD4HOTfedTeP-hzAwfG8C8EuPECMq5H0y5mSvjitNEoJvRNIVA__NsdU3nBDxcCTEQrxLiJcanLRi) | - |  |  |
| 28 |  | Cobalt | Co | [7440-48-4](http://www.baidu.com/link?url=d4SNpmKeK5bu4C5sNEkVXbKxp_RkSKhdkE-LeA3e_O_XH-N8mcX_C0jFvjH6m-PsSe_KSZ__xe680jcnFdeSphbX3UqMtd70Nvzypgej7_y) |  |  |  |
| 29 |  | Nickel | Ni | [7440-02-0](http://www.baidu.com/link?url=gqX5ZwHDdP_NoGYoD99sx-IE2R5zOPvMZKpaJ2cXRn76gxtrirC3fMgweXdVNn9AyZbKxQ3PcvOFSlm8__B-oZCNiE-LWXDKPQthERPwvlq) |  |  |  |
| 30 |  | Gallium | Ga | 7440-55-3 |  |  |  |
| 31 |  | Arsenic | As | 36275-68-0 | 0.1 mg/kg ww Inorganic Arsenic |  |  |
| 32 |  | Selenium | Se | 20405-64-5 | - |  |  |
| 33 |  | Rubidium | Rb | 13446-72-5 |  |  |  |
| 34 |  | Silver | Ag | 7440-22-4 |  |  |  |
| 35 |  | Cadmium | Cd | 7440-43-9 | 0.5 mg/kg ww |  |  |
| 36 |  | Caesium | Cs | 18459-37-5 | - |  |  |
| 37 |  | Barium | Ba | 7440-39-3 | - |  |  |
| 38 |  | Mercury | Hg | 7439-97-6 | 1.0 mg/kg ww |  |  |
| 39 |  | Tin | Tl | 14683-07-9 | - |  |  |
| 40 |  | Lead | Pb | 7439-92-1 | 0.5 mg/kg ww |  |  |
| 41 | Organochlorine pesticides | Alpha-HCH | α-HCH | 319-84-6 | 0.1 mg/kg ww | National food safety standards Maximum residue limits of pesticides in food (GB 2763-2021) (5) | Environment (6) |
| 42 |  | Beta-HCH | β-HCH | 319-85-7 |  |  |  |
| 43 |  | Gama-HCH | γ-HCH | 58-89-9 |  |  |  |
| 44 |  | Delta-HCH | δ-HCH | 319-86-8 |  |  |  |
| 45 |  | 2,4'-DDT | o,p'-DDT | [789-02-6](https://www.chemsrc.com/baike/139354.html) | 0.5 mg/kg ww |  |  |
| 46 |  | 4,4'-DDT | p,p'-DDT | [50-29-3](https://www.chemsrc.com/baike/749899.html) |  |  |  |
| 47 |  | 2,4'-DDD | o,p'-DDD | [102976-58-9](https://www.chemsrc.com/baike/1657179.html) |  |  |  |
| 48 |  | 4,4'-DDD | p,p'-DDD | [72-54-8](https://www.chemsrc.com/baike/1183759.html) |  |  |  |
| 49 |  | 2,4'-DDE | o,p'-DDE | [3424-82-6](https://www.chemsrc.com/baike/195588.html) |  |  |  |
| 50 |  | 4,4'-DDE | p,p'-DDE | 72-55-9 |  |  |  |
| 51 |  | Aldrin | ALD | 309-00-2 | 0.01 mg/kg ww | Positive List System, Japan (7) |  |
| 52 |  | Isodrin | ISO | 465-73-6 | - |  |  |
| 53 |  | Dieldrin | DIE | 60-57-1 |  | - |  |
| 54 |  | Endrin | END | 72-20-8 |  |  |  |
| 55 |  | Heptachlor | HEP | 76-44-8 |  |  |  |
| 56 |  | Heptachlor epoxide | HEPE | 1024-57-3 | 0.01 mg/kg ww | Positive List System, Japan (7) |  |
| 57 |  | cis-Chlordane(alpha) | α-CHL | 5103-71-9 | - |  |  |
| 58 |  | γ- Chlordane | γ-CHL | 5103-74-2 |  |  |  |
| 59 |  | Endosulfan I | END I | 742-93-4 |  |  |  |
| 60 |  | Endosulfan- II | END II | 1031-07-8 | 0.01 mg/kg ww | Positive List System, Japan (7) |  |
| 61 |  | Methoxyl DDT | Me DDT | [72-43-5](https://www.chemsrc.com/baike/669263.html) | - |  |  |
| 62 |  | Hexachlorobenzene | HEX | 118-74-1 | 0.01 mg/kg ww | Positive List System, Japan (7) |  |
| 63 |  | Mirex | MIR | 2385-85-5 | - |  |  |
| 64 | Dioxin | AhR activity Dioxin | Dioxin | [290-67-5](https://www.chemsrc.com/baike/302635.html) | 6.5 TEQ pg/g | EU (8) | Environment (9) |

Table S2 The dietary risk of the residual contaminant in crab is defined as the [superposition](javascript:;) of severity properties and exposure routes. Details containing A: hazard properties; B: toxicity potency (refer to ADI value); C: usage; D: high exposure groups (refer to detection frequency); E: ratio of aquatic product in the diet; F: residue level. The scores attributed to various of risk factors were shown as below.

| Scores | (A)  Hazard properties | (B)  Toxicity potency | (C)  Usage | (D)  High exposure group | (E)  Ratio of aquatic product in diet | (F)  Residue level |
| --- | --- | --- | --- | --- | --- | --- |
| 0 | There are no harmful effects. | ADI: > 10 µg/kg bw/day | The whole aquaculture cycle was in use for <2.5% of the time. | There is no possibility of high exposure, and the detection frequency is less than 2.5%. | < 2.5% of the total diet | No evidence of detectable residue |
| 1 | There are pharmacological harmful effects (increased blood pressure, disturbance of intestinal flora, etc.) but they can be eliminated. | ADI: 1-10 µg/kg bw/day | The whole aquaculture cycle was in use for 2.5%-20% of the time. | There is possibility of high exposure, and the detection frequency is between 2.5% and 20%. | 2.5%-20% of the total diet | Residues detected below MRL |
| 2 | There is organ toxicity (liver damage, kidney damage, etc.), but it can be eliminated. | ADI: 0.1-1 µg/kg bw/day | The whole aquaculture cycle was in use for 20%-50% of the time. | Close to high exposure, the detection frequency is between 20% and 50%. | 20%-50% of the total diet | Residues detected above MRL (< 5% of the samples) |
| 3 | Irritant, which may cause allergy. | ADI: 0.01- 0.1 µg/kg bw/day | The whole aquaculture cycle was in use for >50% of the time. | High exposure group, and the detection frequency is between 50% and 75%. | >50% of the total diet | Residues detected above MRL (> 5% of the samples) |
| 4 | The is no carcinogenic mechanism, but irreversible organ toxicity, embryo toxicity, immune toxicity and so on. | ADI: 0.001-0.01 µg/kg bw/day | - | High exposure group, and the detection frequency is between 75% and 95%. | - | - |
| 5 | There are irreversible neurotoxic, reproductive toxic effects, and can cause mutation. | ADI: 0.0001-0.001 µg/kg bw/day | - | High exposure group, and the detection frequency is more than 95%. | - | - |
| 6 | Carcinogens with a clear carcinogenic mechanism. | ADI: < 0.0001 µg/kg bw/day | - |  | - | - |

Table S3 With the calculation formula, A: hazard properties; B: toxicity potency; C: usage; D: high exposure groups; E: ratio of aquatic product in the diet; F: residue level. The dietary risk was ranked by comparing the TS value of each contaminant. Total scores were shown as below.

| Contaminants | A  Hazard properties | B  Toxicity potency | C  Usage | D  High exposure group | E  Ratio of aquatic product in diet | F  Residue level | Total Scores |
| --- | --- | --- | --- | --- | --- | --- | --- |
| ΣSAs | 4 | 0 | 3 | 2 | 1 | 1 | 24 |
| ΣQUs | 4 | 1 | 3 | 2 | 1 | 2 | 60 |
| Cr | 3 | 1 | 0 | 4 | 1 | 1 | 20 |
| As | 6 | 1 | 0 | 5 | 1 | 1 | 42 |
| Cd | 5 | 1 | 0 | 5 | 1 | 2 | 72 |
| Hg | 5 | 2 | 0 | 5 | 1 | 1 | 42 |
| Pb | 5 | 1 | 0 | 4 | 1 | 2 | 60 |
| ΣHCHs | 6 | 1 | 0 | 4 | 1 | 1 | 35 |
| ΣDDTs | 6 | 1 | 0 | 4 | 1 | 1 | 35 |
| HEX | 6 | 2 | 0 | 3 | 1 | 1 | 32 |
| HEPE | 5 | 2 | 0 | 3 | 1 | 3 | 84 |
| ENDII | 5 | 1 | 0 | 3 | 1 | 1 | 24 |
| Dixon | 6 | 6 | 0 | 5 | 1 | 1 | 72 |

Reference

1. Agriculture Mo. The Announcement (The 235th) of Ministry of Agriculture. China2002. p. 3-.

2. Song C, Li L, Zhang C, Qiu L, Fan L, Wu W, et al. Dietary risk ranking for residual antibiotics in cultured aquatic products around Tai Lake, China. Ecotoxicology and Environmental Safety. 2017;144:252-7.

3. Agriculture Mo. National food safety standards Limits of contaminants in food. China: GB 2762-2012; 2012. p. 1.

4. Nakayama S, Ikenaka Y, Muzandu K, Choongo K, Oroszlany B, Teraoka H, et al. Heavy Metal Accumulation in Lake Sediments, Fish (Oreochromis niloticus and Serranochromis thumbergi), and Crayfish (Cherax quadricarinatus) in Lake Itezhi-tezhi and Lake Kariba, Zambia. Archives of Environmental Contamination and Toxicology. 2010;59(2):291-300.

5. Agriculture Mo. National food safety standards Maximum residue limits of pesticides in food. China: GB 2763-2021; 2021. p. 17-.

6. Pan H, Geng J, Qin Y, Tou F, Zhou J, Liu M, et al. PCBs and OCPs in fish along coastal fisheries in China: Distribution and health risk assessment. Marine Pollution Bulletin. 2016;111(1-2):483-7.

7. Janpan. Positive List System. Japan; 2006.

8. EC. Commission Regulation (EC) No 1881/2006 of 19 December 2006 Setting Maximum Levels for Certain Contaminants in Foodstuffs. Off. J. Eur. Unlon Legis.; 2006. p. 364.

9. Bell JG, Mcghee F, Dick JR, Tocher DR. Dioxin and dioxin-like polychlorinated biphenyls (PCBs) in Scottish farmed salmon (Salmo salar): effects of replacement of dietary marine fish oil with vegetable oils. Aquaculture. 2005;243(1-4):305-14.
